# Implementation of non-communicable disease prevention and control policies in the Western Pacific Region: a 10-year review, 2015–2025

**DOI:** 10.1136/bmjgh-2026-025371

**Published:** 2026-10-14

**Authors:** Azeb Gebresilassie Tesema, Elvis Anyaehiechukwu Okolie, Rohina Joshi

**Affiliations:** 1 School of Population Health, University of New South Wales, Sydney, New South Wales, Australia; 2 The George Institute, Sydney, New South Wales, Australia; 3 Department of Public Health, David Umahi Federal University of Health Sciences State, Uburu, Nigeria

**Keywords:** Health policies and all other topics, Cardiovascular disease, Review

## Abstract

**Background:**

Non-communicable diseases (NCDs) are the leading causes of mortality in the Western Pacific Region. We examined trends in implementation of WHO-recommended policies and their association with country geographic and income groups across the region over the past decade.

**Methods:**

We used data from the five WHO NCD Progress Monitor reports (2015–2025) to analyse aggregate NCD policy implementation across 27 countries. Descriptive statistics were used to analyse policy implementation, while repeated-measures analysis of variance was used to examine trends and differences across the region over the 10-year period and by country classification.

**Results:**

In 2025, the region achieved an average full implementation of 33.5% (SD 17.0) across all NCD policies, with substantial variation between countries and policy domains. Policies related to alcohol advertising restrictions, healthier food reformulation, physical activity campaigns and tobacco taxation showed lower implementation scores. Between 2015 and 2025, average policy implementation improved by 7%. 20 (74%) countries improved their policy implementation scores, with significant gains observed in the Marshall Islands, Nauru, Samoa and Vanuatu. The analysis showed that country classification (by income level, with Pacific Island Countries treated as a distinct geographical grouping) explained approximately 45% of the variation in overall implementation (p<0.001).

**Conclusions and recommendations:**

NCD policy implementation increased modestly over the past decade. On average, nearly half of the WHO-recommended NCD policies remain unimplemented across the region. The implementation gap between Pacific Island Countries and others requires targeted and equitable efforts to accelerate progress across the region.

**WHAT IS ALREADY KNOWN ON THE TOPIC**

- Since 2015, five WHO non-communicable disease (NCD) Progress Monitor reports have assessed the global implementation of recommended NCD policies.
- Existing evidence from one or more WHO monitoring reports published before 2022 indicates substantial variation in NCD policy implementation across countries and income groups.
- However, evidence on temporal trends in NCD policy implementation and associated factors in the WHO Western Pacific Region over the last decade (2015–2025), using all the five Progress Monitor reports, remains limited.

**WHAT THIS STUDY ADDS**

- In 2025, the region fully implemented about one-third of recommended NCD policies, with substantial variation observed between countries and policy domains.
- Between 2015 and 2025, NCD policy implementation showed gradual progress, with an average increase of 7% over the last decade.
- Across the reporting years, most countries improved in policy implementation, while a few regressed.
- Overall implementation remained lower in Pacific Island Countries despite recent gains compared with other groups.

**HOW THIS STUDY MIGHT AFFECT RESEARCH, PRACTICE AND POLICY**

- The findings highlight slow and uneven progress in implementing WHO recommended NCD policies, despite global and regional commitments.
- The results underscore the need for targeted efforts to strengthen policy implementation across the region.
- Although modest progress is evident, persistent disparities between countries indicate that additional support is required to accelerate implementation, particularly in the Pacific Island Countries.

## Introduction

 The Western Pacific Region (WPR) accounts for 25% of worldwide non-communicable disease (NCD) deaths.^1 2^ It is the second populous region with about 1.9 billion people across diverse socioeconomic, demographic, ethnic and health system characteristics.^1–3^ In 2019, the four major NCDs accounted for 12 million deaths, which represented 87% of all deaths within WPR.^1 2^ The region is contending with a rapidly ageing population and shifting health needs, including the rising burden and emergence of NCDs as the leading cause of morbidity and premature mortality due to ongoing epidemiologic, demographic and socioeconomic transitions.^1–3^ Such transition has accelerated the prevalence of NCD risk factors such as alcohol and tobacco use, physical inactivity and unhealthy diets.^3 4^ Although considerable reductions in premature NCD mortality in WPR were observed from 2000 to 2010, these improvements were not sustained and inequities persist within the region.^2 3^ According to the *Lancet*’s NCD Countdown 2030 report, only New Zealand, the Republic of Korea and Singapore, all high-income countries in WPR, are on course to meet the Sustainable Development Goal (SDG 3.4 target of reducing premature NCD mortality.^2 5^

More recently, progress in reducing premature NCD mortality has slowed, stalled or even reversed in a significant proportion of low-income and Pacific Island Countries and Territories (PICTs) within the region.^2 3^ The regional problem called for collective action and a shared vision to reverse the trend.^2 6^ Drawing on commitments in the Political Declaration and the WHO Global Action Plan, the WPR Action Plan for the Prevention and Control of NCDs (2014–2020)—extended to 2030—was developed to reduce NCD-related morbidity, disability and premature mortality through evidence-based interventions.^6 7^ The plan’s adoption of a ‘whole-of-government’ and ‘whole-of-society’ approach reflected a paradigm shift, positioning NCD prevention and control as an integral component of national development.^7^

Similarly, the 2014 Pacific NCD Roadmap provided an investment case for addressing NCD, not just as a health challenge, but also a significant threat to the region’s economic development.^8^ Following the endorsement of the Pacific NCD Roadmap, the Pacific Monitoring Alliance for NCD Action (MANA) was established in 2017 to strengthen accountability among PICTs and track implementation progress.^9 10^ The MANA dashboard has contributed to the production of three progress reports covering NCD policy implementation in PICTs from 2017 to 2022.^9^ Equally notable is the 2023 development of the Regional Action Framework for Non-communicable Disease Prevention and Control in the Western Pacific, which aims to reduce premature mortality due to NCDs, and support sustainable socioeconomic development across the region.^2^ Each country’s health system capacity will have a profound impact on the region’s collective response to the NCD problem.^2^

Leveraging opportunities for evaluation, experience-sharing and learning between countries on policy and health system responses to NCDs is critical.^2 11^ To strengthen accountability for commitments made by member states, WHO has produced NCD Progress Monitor reports over the last 10 years (2015–2025), highlighting each member state’s progress in NCD policy implementation using a set of indicators.^12–16^ As we move closer to the 2030 timeline for achieving the SDGs, the WHO Global Action Plan and the Regional Action Plan, understanding how countries in WPR have fared in translating global and regional policy recommendations into national action is crucial. Additionally, evidence suggests that COVID-19 disrupted NCD prevention and management, highlighting the need to assess whether this is reflected in shifts in NCD policy implementation in the region to identify gaps and inform priorities.^6 17–19^ Although a few global and country-specific assessments based on the NCD Progress Monitor reports have been published previously,^17 18 20 21^ evidence on temporal trends in policy implementation across the WHO WPR over the last decade (2015–2025)—using the five Progress Monitor reports—remains limited. Hence, this study aimed to (1) examine trends in the implementation of the global NCD policy recommendations across the WHO WPR over a 10-year period, (2) assess whether NCD policy implementation is associated with country classification during this period and (3) examine country progress in the most recent (2025) implementation of NCD policies.

## Methods

We assessed the progress of 27 Western Pacific Countries in implementing the WHO-recommended NCD policies using data from WHO NCD Progress Monitor reports published over the last decade (2015–2025). Since the first NCD Progress Monitor report was published in 2015, after its launch at the 2015 UN Sustainable Development Summit, four additional reports have been published in 2017, 2020, 2022 and 2025. The first four NCD Progress Monitor reports included 10 progress indicators, while the 2025 report added an 11th indicator. These indicators included: (1) time-bound national NCD targets, (2) functional systems for reliable mortality data, (3) periodic risk factor surveys, (4) national integrated NCD policy/strategy/action plan, (5) tobacco demand-reduction measures, (6) harmful use of alcohol reduction measures, (7) unhealthy diet reduction measures, (8) public education and awareness campaign on physical activity, (9) guidelines for management of cancer, cardiovascular diseases (CVDs), diabetes and chronic respiratory diseases (CRDs), (10) drug therapy/counselling to prevent heart attacks and strokes (2015–22) and essential medicines and technologies for CRDs (2025) and (11) human papillomavirus (HPV) vaccination programme (2025). Each indicator comprised a varying number of policy measures (hereafter referred to as policies; online supplemental appendix 1 includes the full indicator definitions).^12–16^ These indicators and policy measures constitute the core components of the WHO’s NCD Progress Monitor reports, which promote consistent reporting across Member States using the most recent epidemiological and policy implementation data.^12^ The Progress Monitor reports also include national demographic and mortality data, such as population size, proportions and numbers of deaths from NCDs, and the risk of premature death from the four main NCDs (CVD, cancer, CRD or diabetes).^12^ These data were sourced from multiple WHO databases and other sources; detailed indicator sources and definitions are available in the WHO NCD Progress Monitor reports and its Explanatory Notes.^22^

For this analysis, we extracted policy implementation data for each indicator from the five WHO NCD Progress Monitor reports using a Microsoft Excel spreadsheet for the 27 countries. We also extracted mortality data, specifically the percentage of deaths from NCDs and the risk of premature mortality from target NCDs, for countries with available data in the 2025 report. Mortality data were unavailable for 6 countries with populations below 90 000 (Cook Islands, Marshall Islands, Nauru, Niue, Palau and Tuvalu); hence, mortality data were included for 21 countries. Additionally, country income data were extracted from the 2024 World Bank Group income classification data.^23^

Based on the WHO Explanatory Notes^22^ and previous studies,^20 21 24^ policy indicators and measures were scored as 1 point for fully achieved policies, 0.5 points for partially achieved policies and 0 points for policies that were not achieved or for which implementation data were unavailable. These scores reflect the reported existence or achievement of policy measures at the specified level—full, partial or not achieved—and do not measure implementation effectiveness, enforcement in practice, population coverage, behavioural change or health impact.^21^ Once the data were cleaned and recoded, they were exported into SPSS V.30 for analysis. Consistent with the WHO WPR NCD Country Capacity Survey methodology,^6 7^ countries were classified into high-income countries (HICs), low- and middle-income countries (LMICs) and Pacific Island Countries (PICs). PICs were analysed as a distinct subregional grouping rather than according to World Bank income classifications, reflecting their shared geographical, demographic and health system characteristics.

### Statistical analysis

The statistical analysis involved two components. First, we assessed policy implementation levels across the 27 countries for the most recent reporting period (2025). Next, we conducted a repeated-measures analysis of variance (ANOVA) to evaluate trends and differences in implementation levels over the 10-year period using data from the five Progress Monitor reports (2015, 2017, 2020, 2022 and 2025). Complete data are available for all country–year observations in online supplemental appendix 1.

For the 2025 data, descriptive statistics, including the mean, SD and range, were calculated to assess policy implementation across the 22 policies reported that year across the countries. Implementation score was reported across six thematic areas: (1) Governance and NCD surveillance capacity (indicators 1, 2, 3, 4 and 9), (2) Tobacco control policies (indicators 5a–e), (3) Alcohol control policies (indicators 6a–c), (4) Healthy diet policies (indicators 7a–f), (5) Physical activity policy (indicator 8) and (6) NCD medicines and technologies policies (indicators 10 and 11). We used a bar chart to display variations in the mean implementation of the individual policies. A heat map was used to visualise levels of policy achievement (full, partial or none) across countries.

For the second part, implementation scores were calculated using policies available across all five reporting periods. To enable consistent comparisons over time and avoid artefactual inflation, indicators newly added in the 2025 report or available only in other reporting years were excluded, resulting in 15 policies included in the analysis (online supplemental appendix 1). Aggregate implementation scores and mean percentages were calculated for the 15 NCD policies; excluding policies not included in all reporting years resulted in a maximum possible score of 15 per country across the five reporting years. Consistent with the WHO Progress Monitor reports and previous studies that have analysed its dataset,^18 20 21^ all policy indicators were weighted equally. This approach provides a descriptive summary of reported policy achievement; it does not imply that all measures have equal effectiveness or impact on NCD outcomes. Mean policy implementation scores were calculated by year, income group and policy to examine variations in implementation, and boxplots were used to illustrate these variations. Changes in overall policy implementation between the first reporting year (2015) and the most recent reporting year (2025) were calculated using total implementation scores to assess whether countries improved, regressed or remained unchanged.

A general linear model with repeated-measures ANOVA was used to evaluate temporal trends and between-time-point differences in policy implementation across the region over the 10-year period and across country classification. The choice of a repeated-measures ANOVA was based on data for each country’s policy implementation levels being collected at different time points, accounting for correlation within and between countries over time.^25^ Mauchly’s test of sphericity indicated that the assumption of sphericity was violated (p<0.001); therefore, the Greenhouse–Geisser correction was applied. Within-subjects effects, between-subjects effects and pairwise comparisons were conducted, and results were reported using *F*-statistics with p values and effect size estimates.

### Patient and public involvement

Patients and the public were not involved in this study.

## Results

### Study countries and coverage of policy indicators

Of the 27 countries, 13 (48%) are PICs and the remaining 14 (52%) are distributed equally between LMICs and HICs. Among the 21 countries with available mortality data, NCDs accounted for an average of 74.7% of total deaths, with substantial between-country variation ranging between 48% (Papua New Guinea) to 91% (China). The mean probability of dying between ages 30 and 70 from major NCDs was 24.3%, ranging from 7% (Republic of Korea) to 44% (Kiribati); with a higher proportion among PICs (36.1%; SD+6.2%) and a lower proportion in HICs (9.7%; SD±2.9%). The findings are organised into two main sections. The first presents policy implementation in 2025, while the second section examines policy implementation across the five monitoring periods (2015, 2017, 2020, 2022, and 2025).

### Status of NCD policy implementation in 2025

In 2025, the implementation of NCD policies across the 27 countries showed variation across policy indicators.

#### Governance and NCD surveillance capacity policies (indicators 1, 2, 3, 4 and 9)

In the 2025 report, more than half of the countries (16, 59.3%) had fully achieved the indicator by setting time-bound national targets based on the WHO guidance. Seven (26%) countries had a functioning system for generating reliable cause-specific mortality data on a routine basis, and 10 (37%) had conducted a WHO STEPwise approach to NCD risk factor surveillance survey (STEPS survey) every 5 years, as well as a national health survey for NCD risk factor surveillance using self-reported questionnaires and physical and biological measures. 14 (56%) countries reported fully operational multisectoral national policies, strategies or action plans integrating the major NCDs and their shared risk factors. Over half of the countries (51.9%) reported only partial implementation of evidence-based national guidelines or protocols for the management of major NCDs through a government-recognised primary care approach (figure 1 - Heat Map).

**Figure 1:**
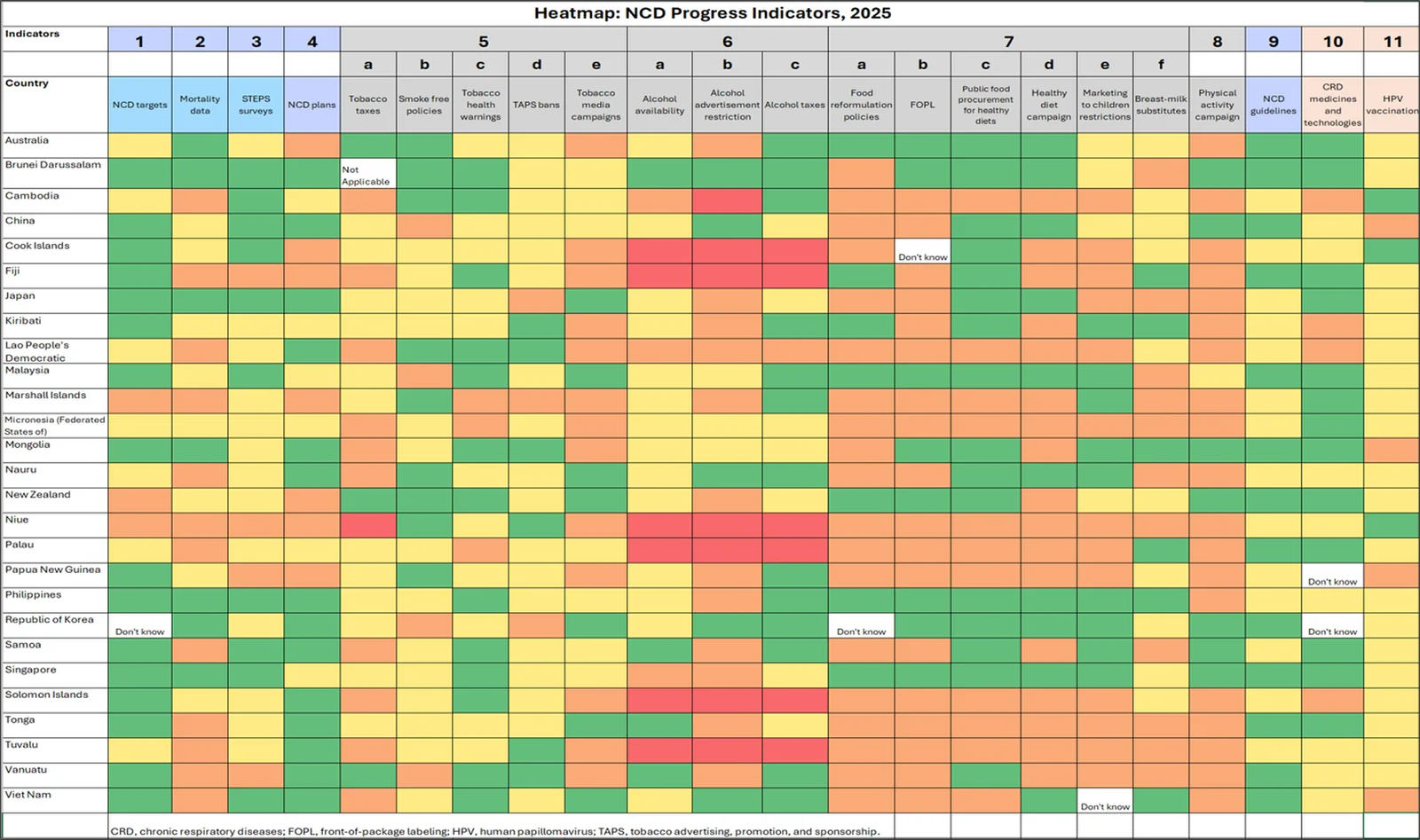
Heatmap of progress in implementing NCD policies across countries in the WHO Western Pacific Region, 2026. 
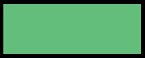
, Fully achieved; 
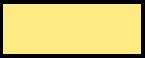
, Partially achieved; 
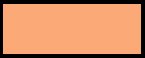
, Not achieved; 
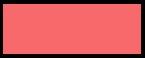
, No data. BCC, Behaviour Change Communication; NCD, non-communicable disease; STEPS, WHO STEPwise approach to noncommunicable disease (NCD) risk factor surveillance.

#### Tobacco control policies (indicators 5a–e)

Tobacco control policies were among the least implemented measures in 2025. Three countries (Australia, New Zealand and Vanuatu) fully achieved tobacco taxation policy. Policies on secondhand tobacco smoke legislation and health warning requirements were fully implemented by 9 (33.3%) and 13 (48.1%) of the countries, respectively, whereas less than a quarter of the counties fully implemented policies on bans on advertising, promotion and sponsorship (6 (22%)); and media campaigns to inform about the harms of tobacco use (7 (26%)).

#### Alcohol control policies (indicators 6a–c)

Progress on implementing policies to address the harmful use of alcohol has been minimal in most countries. While 13 (48%) of the countries fully achieved policy on increasing excise taxes on alcoholic beverages, four countries (Brunei Darussalam, Samoa, Tonga and Vanuatu) had fully enacted restrictions on the physical availability of retailed alcohol, and five countries (Brunei Darussalam, China, Nauru, Republic of Korea and Viet Nam) had fully enacted restrictions on exposure to alcohol advertising.

#### Healthy diet policies (indicators 7a–f)

Diet-related policies showed considerable gaps in 2025. Although more than half of the countries fully implemented healthy eating policies for public food procurement, 7 (26%) and 8 (30%) fully implemented reformulation policies for healthier food and beverage products and front-of-package labelling, respectively. Behaviour changes communication and mass media campaigns for healthy eating have been fully implemented in 10 (37%) countries, and recommendations on marketing unhealthy foods to children have been fully implemented in 9 (33.3%) countries. Implementation of the International Code of Marketing of Breast-milk Substitutes remained limited, with most countries (77.7%) either not implementing or partially implementing the policy.

#### Physical activity policy (indicator 8)

Full implementation of the policy supporting campaigns to promote physical activity was reported in 7 (26%) countries, while 19 (70%) countries had either not implemented the policy or had no available data.

#### NCD medicines and technologies policies (indicators 10 and 11)

In 13 (48.1%) countries, full implementation of essential medicines and technologies for CRDs was generally available in public primary care facilities; 3 countries (Cambodia, Cook Islands and Niue) fully reported the implementation of an HPV vaccination programme (figure 1).

Comparing individual policy implementation in the region, the average implementation scores—accounting for both full and partial implementation—varied considerably across policies. Policy related to setting time-bound national targets and implementing evidence-based national guidelines or protocols for the management of major NCDs demonstrated the highest average implementation, with mean scores of 74% and 72%, respectively. Alcohol-related and tobacco-related measures showed lower mean scores (figure 2).

**Figure 2:**
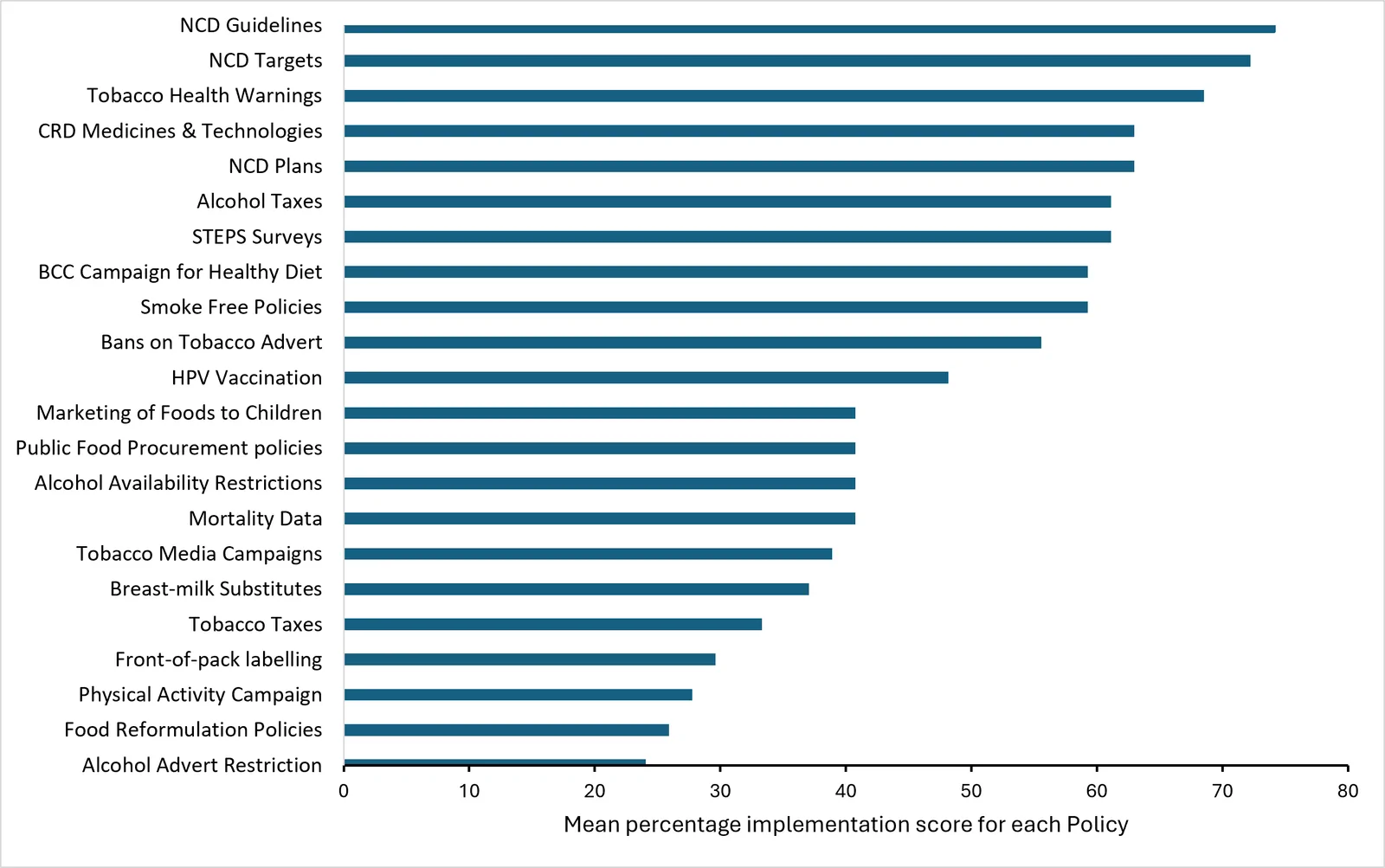
Mean implementation score for each NCD policy in the WHO Western Pacific Region, 2025. BCC, Behaviour Change Communication; CRD, chronic respiratory disease; HPV, human papillomavirus; NCD, non-communicable disease; STEPS, WHO STEPwise approach to noncommunicable disease (NCD) risk factor surveillance.

Overall, in 2025, the WHO WPR achieved an average full implementation of 33.5% (SD 17.0) across the 22 NCD policies, with substantial variation observed between countries, ranging from 4.5% in Micronesia (Federated States of) to 68.2% in Brunei Darussalam. Figure 1 highlights the implementation of policies across countries.

### Non-communicable disease policy implementation from 2015 to 2025

#### Has the overall NCD policy implementation changed over time in the WHO WPR?

Using the aggregate policy scores for the 15 NCD policies consistently reported across the five assessment years, mean implementation was 43.6% (SD 18.7) in 2015, 45.9% (SD 15.3) in 2017, 48.4% (SD 13.4) in 2020, 46.3% (SD 13.8) in 2022 and 50.9% (SD 15.7) in 2025. Although considerable variation was observed across countries in each round, overall policy implementation showed a gradual increase over time, except for a slight decline in 2022 (figure 3a). Across the years, policy implementation was lower among PICs with an overall mean of 37.4% (SD 12.1), compared with HICs (56%, SD 12.5) and LMICs (55.8%, SD 13.1) (figure 3b).

**Figure 3:**
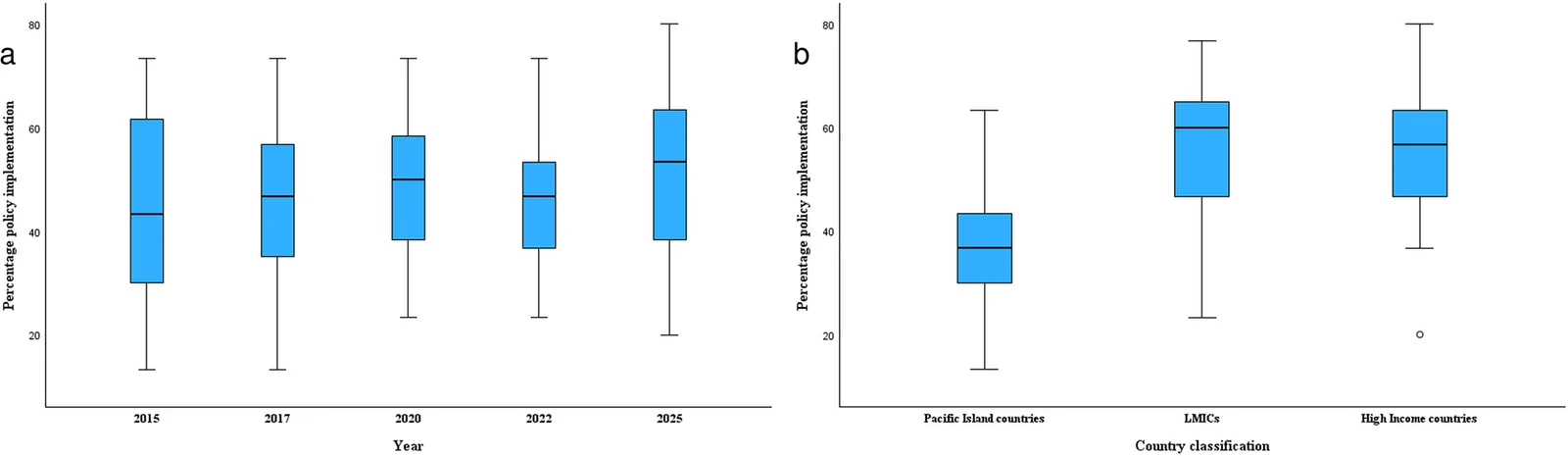
Box plots (**a**) percentage of policy implementation from 2015 to 2025 and (**b**) percentage of policy implementation across country income groups from 2015 to 2025. LMICs, low- and middle-income countries.

Between 2015 and 2025, the overall policy implementation score showed mixed results across the countries. 20 (74%) countries improved their policy implementation scores, with significant gains observed in the Marshall Islands, Nauru, Samoa and Vanuatu. However, five (Australia, Fiji, Malaysia, New Zealand and Tuvalu) regressed, and Japan and Niue remained unchanged. Percentage change highlights substantial proportional gains in countries with few policies in 2015, with some (eg, Samoa) achieving over a 100% increase (table 1).

**Table 1:** NCD policy implementation scores and changes in the Western Pacific Region, 2015–2025

Country	Total policy implementation score (N=15) in 2015	Total policy implementation score in 2025	Absolute change in total policy implementation 2025–2015	Relative change in percentage 2025–2015 (%)
Australia	9.5	8.5	−1.0	−10.5
Brunei Darussalam	11.0	12.0	1.0	9.1
Cambodia	5.5	6.5	1.0	18.2
China	8.5	10.0	1.5	17.7
Cook Islands	4.5	5.5	1.0	22.22
Fiji	9.5	5.0	−4.5	−47.4
Japan	7.0	7.0	0.0	0.0
Kiribati	6.5	9.0	2.5	38.5
Lao People’s Democratic Republic	3.5	6.0	2.5	71.4
Malaysia	10.0	9.5	−0.5	−5.0
Marshall Islands	2.0	5.0	3.0	150.0
Micronesia (Federated States of)	4.5	5.0	0.5	11.1
Mongolia	10.0	11.5	1.5	15.0
Nauru	3.0	8.0	5.0	166.7
New Zealand	9.0	8.5	−0.5	−5.6
Niue	3.0	3.0	0.0	0.0
Palau	4.5	5.0	0.5	11.1
Papua New Guinea	5.5	6.5	1.0	18.2
Philippines	9.5	10.5	1.0	10.5
Republic of Korea	8.5	9.5	1.0	11.8
Samoa	2.0	9.5	7.5	375.0
Singapore	9.5	10.0	0.5	5.4
Solomon Islands	4.5	6.0	1.5	33.3
Tonga	6.5	7.0	0.5	7.7
Tuvalu	7.5	4.5	−3.0	−40.0
Vanuatu	3.0	8.0	5.0	166.7

BCC, Behaviour Change Communication; NCD, non-communicable disease; STEPS, WHO STEPwise approach to noncommunicable disease (NCD) risk factor surveillance.

Further analysis of policy implementation in the region over the 10-year period, using a repeated-measures ANOVA, indicated that implementation improved over this period, with a significant within-subjects effect of year (Greenhouse-Geisser: *F*(2.36, 61.36) = 3.57, p=0.02, partial η² = 0.12). Additionally, pairwise comparisons showed an overall increase in policy implementation, with the largest increase observed from 2015 to 2025 (mean difference=−7.28, p=0.02), but improvements continued in subsequent reporting years. Although there was decline between 2020 and 2022 (mean difference=2.09), this change was not statistically significant. The implementation increased again from 2022 to 2025 (online supplemental appendix 2).

When country classification was included as a factor, the overall within-subjects effect of year became marginally significant (*F*(2.22, 53.36) = 3.03, p=0.052, partial η² = 0.112), suggesting modest changes in policy implementation over time. There was no significant interaction between year and country classification (*F*(4.45, 53.36) = 0.69, p=0.616, partial η² = 0.054), indicating that the pattern of improvement over time was generally consistent across countries. However, country classification showed a significant between-subjects effect on overall policy implementation (*F*(2, 24) = 10.03, p<0.001, partial η² = 0.455), reflecting differences in implementation across country groups and accounting for approximately 45% of the variance (online supplemental appendix 2).

## Discussion

This study used data from the NCD Progress Monitor reports (2015–2025) to examine progress in NCD policy implementation across the WHO WPR over the 10-year period. Overall, average policy implementation improved by 7% over the last decade, increasing from 43.6% in 2015 to 50.9% in 2025, with marginal variation across the reporting years. There was substantial variation between countries and across policy domains over the past decade. In 2025, the region achieved an average of 33.5% full implementation of the WHO-recommended NCD policies, with substantial variation between countries and policy areas. Policy related to evidence-based NCD guidelines, setting time-bound national targets, graphic warnings on tobacco packaging and availability of essential medicines for CRDs were among the most implemented policies in 2025. However, policies such as restrictions on alcohol advertising, reformulation of policies for healthier food, physical activity campaigns, and tobacco taxation showed lower implementation scores.

At the regional level, overall NCD policy implementation progressed slowly over the past decade. Although these gains are commendable, the modest increase indicates that, on average, nearly half of the WHO-recommended NCD policies remain unimplemented across the region. This finding is not peculiar to the region, as Allen *et al* reported in their global analysis of NCD policy implementation that progress has stagnated, with a 2020 implementation score of 47%.^24^ Nevertheless, the observed improvements align with sustained regional and global efforts to strengthen NCD prevention and control, including the WHO Global Action Plan for the Prevention and Control of NCDs and the Regional Action Framework for Non-communicable Disease Prevention and Control in the Western Pacific.^2 26^

Despite progress, we observed a statistically insignificant decline in average implementation score in 2022 (46.3%) compared with 2020 (48.4%). This decline coincided with the COVID-19 pandemic and likely reflects documented disruptions to health systems, including NCD services, and weaknesses in NCD policy prioritisation during the pandemic.^24 27^ Lessons from COVID-19 reinforce the perspective that siloed health system approaches are more vulnerable to disruptions and the need to integrate NCD care across the health system, especially in the primary healthcare system.^28^

The repeated-measures ANOVA showed that country classification (by income level, with PICs treated as a distinct geographical grouping) explained approximately 45% of the variation in overall implementation. This is consistent with the descriptive findings of lower average policy implementation in PICs (37.4%) compared with LMICs (55.8%) and HICs (56%). This pattern is partly explained by differences in baseline policy implementation levels across country groups in 2015; PICs started from lower levels despite substantial absolute gains in subsequent years (eg, Samoa, Nauru, Vanuatu and the Marshall Islands) (table 1). Additionally, the implementation gap between PICs and others mirrors disparities in NCD mortality, risk factor prevalence, socioeconomic status, and the limited health system response.^1 29^

The association between national country classification and NCD policy implementation observed in this study aligns with prior evidence highlighting the importance of financial capacity and human capital in translating global policy recommendations into sustained national action.^18 21^ Despite the importance of these resources for policy implementation, this study demonstrates that income level alone may not always be the primary determinant, as some LMICs and PICs achieved implementation levels similar to those of HICs. This is consistent with previous studies indicating that income level may not be the main determinant of NCD policy implementation, as several policies are cost-effective and some generate revenue.^4 24 26^ Beyond a country’s income level, other determinants such as political commitment, health systems capacity and development assistance, especially for PICs and LMICs, are critical for policy implementation.^2 3^ Nevertheless, addressing existing inequities—particularly for PICs—requires careful consideration of country-specific challenges, including a high NCD burden, geographical and logistical constraints and resource limitations that hinder the health system’s capacity to respond effectively to NCDs.^29 30^

At the country level, between 2015 and 2025, policy implementation significantly increased in Samoa, Nauru, Vanuatu and the Marshall Islands. In contrast, countries such as Fiji, Tuvalu, Australia and New Zealand regressed in their policy implementation scores during the same period, while Japan and Niue remained unchanged. These trends indicate that progress in NCD policy implementation is non-linear and that prior performance does not ensure sustained implementation over time.^12 16 18^ This finding underscores the importance of examining country-level patterns, as regional-level trends may mask significant country-level performance that warrants critical attention.

In 2025, average full implementation across the region remained suboptimal (33.5%), with continued variation between countries, ranging from 4.5% in Micronesia (Federated States of) to 68.2% in Brunei Darussalam. The study also identified substantial variation across policy domains, reflecting differing levels and trajectories of implementation across countries in 2025. Policies related to paper-based NCD guidelines, national targets and clinical service interventions were implemented more widely across countries than population-level policies addressing unhealthy diets, tobacco use, alcohol consumption and physical inactivity. For instance, only three countries (Australia, New Zealand and Vanuatu) fully achieved tobacco taxation policy in 2025. Similarly, fewer than six countries had fully implemented policies on restricting the physical availability of alcohol and exposure to alcohol advertising. Similar patterns were observed in the WHO Region of the Americas.^31^ These findings align with global evidence showing that upstream regulatory interventions are more challenging to implement due to political and commercial determinants of health, which require more complex cross-sectoral coordination.^32^ Moreover, coordinated industry political activities may hinder the adoption and implementation of effective NCD policies, particularly regulatory measures.^33^ While the WHO Framework Convention on Tobacco Control influences the tobacco control policy, enforcement and taxation gaps persist in several countries.^34^

The 2025 implementation levels for diet and physical activity policies call for attention as they were the domains that showed some of the widest gaps in implementation, with about one-third or less of the countries reporting full implementation of reformulation of policies for healthier food and beverage products (26%), front-of-package labelling (30%) and physical activity campaigns (26%). This finding may reflect the considerable use of ‘soft’ policy instruments such as campaigns, partnerships and recommendations for nutrition and physical activity policies compared with the use of ‘hard’ policy instruments such as regulatory and fiscal instruments for tobacco and alcohol control policies, which possess a higher degree of enforcement.^35^ Equally important is the intersection of nutrition policies with trade, agriculture and commerce, which creates multilevel competing priorities that impede regulatory actions within countries.^36^ Closing implementation gaps in these domains will require an audit and a rethinking of current strategies, including the adoption of evidence-based, cost-effective and sustainable approaches.

Despite the importance of data in shaping NCD policy and programmatic decisions, less than 40% of the countries in the region had functioning systems for reliable cause-specific mortality data and had conducted the WHO STEPS survey as recommended. Gaps in STEPS implementation in some countries may partly reflect reliance on alternative national surveys and routine data systems to inform NCD prevention and control. However, implementation gaps may also stem from resource constraints and national capacity limitations. Strengthening NCD surveillance systems remains essential to inform NCD policies and track trends in risk factors.^37^ Notwithstanding this, measurable progress was observed in the implementation of evidence-based national guidelines for the management of major NCDs through a primary care approach, with more than half of the countries in the region fully implementing this policy.

### Limitations of the study

This study has some limitations. First, it relies on data from the WHO NCD Progress Monitor reports; therefore, any methodological constraints or reporting limitations inherent in these data may have influenced the findings. This limitation reflects a constraint of using the available Progress Monitor dataset, which does not provide a specific weighting framework based on the relative contribution or potential impact of individual policy indicators. Because the effectiveness and potential contribution of policy measures may vary, equal weighting may understate the contribution of higher-impact measures and overstate that of lower-impact measures.^18 21^ Second, although policy availability and higher implementation scores are indicative of national commitment to NCD prevention and control, they do not necessarily reflect the extent to which policies are enforced, adequately address population needs, reach underserved and structurally marginalised population groups or reduce NCD-related morbidity and mortality. Conversely, countries reporting partial or no achievement may still be addressing the NCD burden through alternative mechanisms not captured in the monitoring framework (eg, strong routine health information systems in place of STEPS surveys). Additionally, as the WHO NCD Progress Monitor reports data are quantitative, we may not fully capture contextual factors or ongoing alternative initiatives in our analysis. Future studies would benefit from complementing these quantitative assessments with qualitative analyses and country validation processes to understand country-specific contexts and implementation pathways better. Despite these limitations, the study provides a comprehensive regional overview of NCD policy implementation trends.

### Policy implications

This study highlights the need for targeted and equitable efforts to strengthen NCD policy implementation across the region. While slight progress is evident, persistent disparities across countries and policy domains indicate that additional support is required to accelerate implementation, particularly in PICs. Accelerating progress will require stronger implementation of population-level risk-reduction policies, strengthened multisectoral governance and regulatory approaches to address key commercial determinants of NCDs.^18 30^ A whole of government approach is important to effectively address the upstream determinants of NCD-related risk factors.

Drawing from the Regional Action Framework, implementation in the region should also be supported by sustainable financing, cross-sectoral coordination, evidence-based policy and community engagement tailored to each country’s context.^2^ Moreover, improving monitoring, evaluation and accountability systems is critical to track progress and ensure that policies translate into measurable health outcomes. Regional initiatives such as the Pacific MANA contribute by strengthening accountability, monitoring progress and facilitating cross-country learning among PICs.^9 10^ Additionally, strengthening primary health care-based NCD services through the implementation of the WHO Package of Essential Non-communicable Disease Interventions is a key mechanism to support prevention and long-term management of NCD and the achievement of universal health coverage.^2 24^

## Conclusion

Over a 10-year period, NCD policy implementation in the WHO WPR has progressed slowly, with nearly half of recommended policies remaining unimplemented across the region. Substantial variation between countries and across policy domains was observed during this time. Although PICs showed substantial gains in recent years, progress remained constrained by persistent challenges. Addressing these implementation gaps will require targeted and equitable efforts that consider context-specific barriers, including high NCD burden, geographical constraints and resource limitations. As 2030 approaches, deliberate efforts to accelerate implementation are needed to keep the region on track to achieve the Regional Action Plan and the SDG NCD targets.

## Supplementary material

10.1136/bmjgh-2026-025371 online supplemental file 1

10.1136/bmjgh-2026-025371 online supplemental file 2

## Data Availability

Data are available in a public, open access repository. All data relevant to the study are included in the article or uploaded as supplementary information.
