# Supplementary material for: Implementation of non-communicable disease prevention and control policies in the Western Pacific Region: a 10-year review, 2015–2025
Source: BMJ Glob Health. 2026 Oct 14;11(10):e025371. doi: 10.1136/bmjgh-2026-025371 (PMC13691841; doi:10.1136/bmjgh-2026-025371)
Supplement: Supplementary file 1 [file bmjgh-11-10-s002.pdf]

Appendix 2

Table A: Effect of year and country classification on policy implementation, 2015–2025

Tests of Within-Subjects Effects						
**Source**		**df**	**Mean Square**	**F**	**Sig.**	**Partial Eta Squared**
Year	Sphericity Assumed	4	203.992	3.572	.009	.121
	Greenhouse-Geisser	2.360	345.725	3.572	.027	.121
Error (Year)	Sphericity Assumed	104	57.111			
	Greenhouse-Geisser	61.364	96.792			
**Tests of Within-Subjects Effects**						
**Source**		**df**	**Mean Square**	**F**	**Sig.**	**Partial Eta Squared**
Year	Sphericity Assumed	4	177.083	3.027	.021	.112
	Greenhouse-Geisser	2.223	318.587	3.027	.052	.112
Year * country classification	Sphericity Assumed	8	40.403	.691	.699	.054
	Greenhouse-Geisser	4.447	72.689	.691	.616	.054
Error (Year)	Sphericity Assumed	96	58.504			
	Greenhouse-Geisser	53.361	105.253			

Table B: Effect of country classification on policy implementation, 2015–2025

Tests of Between-Subjects Effects						
Source	Type III Sum of Squares	df	Mean Square	F	Sig.	Partial Eta Squared
Intercept	307113.911	1	307113.911	535.913	<.001	.957
Country classification	11496.933	2	5748.467	10.031	<.001	.455
Error	13753.602	24	573.067			

Table C: Pairwise comparisons of overall policy implementation across years, 2015–2025

(I) Year	(J) Year	Mean Difference (I-J)	Std. Error	Sig.[b]	95% Confidence Interval for Difference[b]	
					Lower Bound	Upper Bound
1 (2015)	2	-2.346	2.565	.369	-7.618	2.926
	3	-4.815	2.442	.059	-9.834	.204
	4	-2.716	2.554	.297	-7.966	2.533
	5	-7.284*	2.988	.022	-13.426	-1.142
2 (2017)	1	2.346	2.565	.369	-2.926	7.618
	3	-2.469	1.577	.130	-5.711	.773
	4	-.370	1.544	.812	-3.545	2.804
	5	-4.938*	1.852	.013	-8.745	-1.131
3 (2020)	1	4.815	2.442	.059	-.204	9.834
	2	2.469	1.577	.130	-.773	5.711
	4	2.099	1.127	.074	-.219	4.416

	5	-2.469	1.607	.137	-5.773	.834
4 (2022)	1	2.716	2.554	.297	-2.533	7.966
	2	.370	1.544	.812	-2.804	3.545
	3	-2.099	1.127	.074	-4.416	.219
	5	-4.568*	1.469	.004	-7.587	-1.549
5 (2025)	1	7.284*	2.988	.022	1.142	13.426
	2	4.938*	1.852	.013	1.131	8.745
	3	2.469	1.607	.137	-.834	5.773
	4	4.568*	1.469	.004	1.549	7.587

Based on estimated marginal means

*. The mean difference is significant at the .05 level.

b. Adjustment for multiple comparisons: Least Significant Difference (equivalent to no adjustments).
